# Evaluation of the Increased Genetic Resolution and Utility for Source Tracking of a Recently Developed Method for Genotyping *Cyclospora cayetanensis*

**DOI:** 10.3390/microorganisms12050848

**Published:** 2024-04-24

**Authors:** Susan R. Leonard, Mark K. Mammel, Sonia Almeria, Solomon T. Gebru, David K. Jacobson, Anna C. Peterson, Joel L. N. Barratt, Steven M. Musser

**Affiliations:** 1 Office of Applied Research and Safety Assessment, Center for Food Safety and Applied Nutrition, U.S. Food and Drug Administration, Laurel, MD 20708, USA; mark.mammel@fda.hhs.gov (M.K.M.); maria.almeria@fda.hhs.gov (S.A.); solomon.gebru@fda.hhs.gov (S.T.G.); 2 Division of Parasitic Diseases and Malaria, National Center for Emerging and Zoonotic Infectious Diseases, Centers for Disease Control and Prevention, Atlanta, GA 30329, USA; quh7@cdc.gov (D.K.J.); yyi6@cdc.gov (A.C.P.); nsk9@cdc.gov (J.L.N.B.); 3 Office of the Center Director, Center for Food Safety and Applied Nutrition, U.S. Food and Drug Administration, College Park, MD 20740, USA; steven.musser@fda.hhs.gov

**Keywords:** cyclosporiasis, genotyping, foodborne parasite, targeted amplicon sequencing, traceback, epidemiology, genetic relatedness

## Abstract

*Cyclospora cayetanensis* is a foodborne parasite that causes cyclosporiasis, an enteric illness in humans. Genotyping methods are used to genetically discriminate between specimens from cyclosporiasis cases and can complement source attribution investigations if the method is sufficiently sensitive for application to food items. A very sensitive targeted amplicon sequencing (TAS) assay for genotyping *C. cayetanensis* encompassing 52 loci was recently designed. In this study, we analyzed 66 genetically diverse clinical specimens to assess the change in phylogenetic resolution between the TAS assay and a currently employed eight-marker scheme. Of the 52 markers, ≥50 were successfully haplotyped for all specimens, and these results were used to generate a hierarchical cluster dendrogram. Using a previously described statistical approach to dissect hierarchical trees, the 66 specimens resolved into 24 and 27 distinct genetic clusters for the TAS and an 8-loci scheme, respectively. Although the specimen composition of 15 clusters was identical, there were substantial differences between the two dendrograms, highlighting the importance of both inclusion of additional genome coverage and choice of loci to target for genotyping. To evaluate the ability to genetically link contaminated food samples with clinical specimens, *C. cayetanensis* was genotyped from DNA extracted from raspberries inoculated with fecal specimens. The contaminated raspberry samples were assigned to clusters with the corresponding clinical specimen, demonstrating the utility of the TAS assay for traceback efforts.

## 1. Introduction

Cyclosporiasis, an enteric illness in humans, is caused by infection of the small intestine by the parasite *Cyclospora cayetanensis* [1,2,3]. Throughout this work, any mention of *C. cayetanensis* is inclusive of all three of the newly proposed *Cyclospora* species that cause human illness [4]. Cases of cyclosporiasis in the United States in recent years have encompassed thousands of illnesses, approximately 7.0% of which required hospitalization (CDC—Cyclosporiasis—Outbreaks and Updates, https://www.cdc.gov/parasites/cyclosporiasis/outbreaks/index.html, accessed 5 January 2024). There were over 2000 laboratory confirmed cases in 2018, 2019, and 2023 and over 1000 cases each year from 2020 to 2022. Individuals with cyclosporiasis shed oocysts in their feces, and the parasite is transmitted via the fecal–oral route [3,5,6,7]. However, rather than via direct person-to-person transmission, since the oocysts need time in the environment to become sporulated and infectious, transmission is mainly through the ingestion of food contaminated with feces containing sporulated *C. cayetanensis* oocysts. Infection with *C. cayetanensis* has been epidemiologically associated with the consumption of a variety of contaminated fresh produce items, particularly berries, leafy greens, and herbs [8,9,10,11].

There are no known methods of propagating *C. cayetanensis* in the laboratory, and it is time-consuming and difficult to isolate oocysts from clinical specimens; thus, whole genome sequencing is not a practical option for determining genetic linkages among specimens [12,13]. The U.S. Centers for Disease Control and Prevention (CDC) has been implementing a multi-locus sequence typing (MLST) scheme to genetically cluster *C. cayetanensis* clinical fecal specimens to supplement epidemiologic information when investigating outbreaks [14]. The CDC’s MLST genotyping protocol involves performing conventional PCR targeting eight loci in separate reactions, followed by sequence library preparation and next-generation sequencing of the pooled PCR amplicons. The eight discriminatory targets, two located in the mitochondrial genome and six in the nuclear genome, were previously described [15,16,17]. The eight loci MLST genotyping scheme has been applied to fecal specimens associated with major *C. cayetanensis* outbreaks in the United States [18,19,20] and cyclosporiasis illnesses in Canada [21]. Although this approach has been useful, it has been recognized that the genetic discriminatory power is limited and that additional targets containing phylogenetically informative single-nucleotide polymorphisms (SNPs) are needed to better resolve genetic clusters [21,22,23].

Despite the fact that specific food items have been epidemiologically implicated as vehicles of cyclosporiasis outbreaks, no definitive genetic linkages have been demonstrated between food items and clinical specimens. Contamination levels on fresh produce are very low, and the ability to genotype *C. cayetanensis* on contaminated fresh produce using nuclear markers was not possible until recently, with the development of a very sensitive targeted amplicon sequencing (TAS) method [24]. The exceptional sensitivity was obtained through the addition of a bait-capture step after the target-specific amplification. However, for many clinical fecal specimens, the extra enrichment step may not be necessary due to normally higher parasite loads in clinical compared to food and environmental samples. Along with the increased sensitivity for genotyping *C. cayetanensis*, the TAS assay addressed the need to increase the number of informative genomic targets, expanding the number of loci targeted to 52, of which 49 are located in the nuclear genome. Although the sensitivity of the genotyping assay was previously evaluated [24], the change in phylogenetic resolution and potential changes in placement of samples within genetic clusters resulting from the increased number of informative targets were not fully explored.

In addition to the placement of samples within a hierarchical cluster dendrogram, determination of the optimal number of partitions in which to dissect the dendrogram is important. Ideally, to inform a common source for traceback efforts, clusters should comprise clinical specimens and/or fresh produce samples positive for *C. cayetanensis* that contain the same or very closely related strains, but not other strains. Interpretation of whole genome sequence analyses of bacteria for use in source attribution and outbreak investigations is challenging and includes incorporating several types of information [25]. Due to the diploid nature of the *C. cayetanensis* genome, interpretation of whether two strains arose from the same source of contamination using genome sequence results is even more complex. A framework for selecting the appropriate number of partitions for dendrograms based on *C. cayetanensis* haplotype results has been reported [26]. This framework was developed and tested using a large set of clinical specimens from cases of cyclosporiasis, along with the corresponding epidemiologic information. Application of the framework to include contaminated food samples, particularly with missing haplotype results for some markers in a genotyping scheme, would aid in informing areas for modification in the bioinformatic approach and interpretation for source tracking.

In this study, we assessed the genetic clustering resolution of our recently developed *C. cayetanensis* TAS genotyping assay [24] using a set of 66 clinical specimens. The resolution and accuracy of clustering the fecal specimens was compared to that resulting from the eight loci MLST method. Using raspberry samples inoculated with fecal specimen, the effectiveness of the genotyping assay for traceback investigations was evaluated. We also assessed the performance of the TAS method without the bait-capture enrichment step on a subset of the clinical specimens.

## 2. Materials and Methods

### 2.1. Clinical Specimens

Sixty-six clinical fecal specimens from humans diagnosed with cyclosporiasis were kindly supplied by the CDC and included in this study. The specimens were de-identified, given a unique identifier upon receipt at the CDC, and renamed at the U.S. FDA for use in this study. The selected specimens were collected from 2018 to 2022 and included a subset of 12 specimens with known epidemiologic linkage and many with no known source linkage (Table 1). Additional information related to epidemiologic information terminology can be found in previous reports [18,27]. While the majority of the specimens were preserved in either Cary–Blair or Total Fix transport medium, two were preserved in Para-Pak C&S medium, and eight had either no preservative or the preservative was unknown. The parasite load in the specimens varied, as determined using a previously described real-time PCR assay targeting the 18S rRNA gene [28], with C_T_ values ranging from 18.17 to 31.76 (Table 1). Genotyping had been performed using the CDC’s eight loci MLST scheme, and all eight markers were haplotyped for 52 of the specimens. For the remaining specimens, nine, three, one, and one had seven, six, five, and four markers haplotyped, respectively.

### 2.2. Raspberry Sample Preparation

Raspberries were purchased from a local retail store, divided into 50 g portions, and inoculated with selected clinical fecal specimens, namely either CDC04, CDC16, or CDC43. For inoculation, the fecal specimens were carefully homogenized via a vortex, and approximately 50 µL were collected with a wide-bore pipette. For each sample, 50 µL of a 1:10 dilution and 5 µL of a 1:100 dilution were prepared in sterile water. The 50 g portions of raspberries were inoculated with fecal material by applying the 50 µL of the undiluted and 1:10 dilutions in 10 to 20 droplets spread randomly over the surfaces of the separate raspberry samples. For the 1:100 dilution, 5 µL was spread randomly over the raspberry samples. A control sample receiving no fecal material was included. After inoculation, the samples were allowed to air-dry uncovered at 21 °C for approximately two hours. The samples were then transferred to BagPage filter bags (Interscience Lab, Woburn, MA, USA), sealed with binder clips, and held at 4 °C for 48–72 h before initiating the produce wash step for the fresh berry samples according to the FDA BAM Chapter 19b method as described previously [29].

### 2.3. DNA Extraction and Real-Time PCR

DNA was extracted from the pellets obtained from 200 µL aliquots of the fecal specimens and the pellets obtained from raspberry washes using the FASTDNA SPIN Kit for soil, along with bead-beating using the FastPrep-24 instrument (MP Biomedicals, Santa Ana, CA, USA). The manufacturer’s protocol was followed with modifications as previously described [30]. Further purification was performed for some specimens using the QIAquick PCR Purification Kit (Qiagen, Germantown, MD, USA). DNA concentrations obtained ranged from 1.46 to 600 ng/µL, with an average of 127 ng/µL. Real-time PCR was performed in a duplex reaction targeting both a location in the *C. cayetanensis* mitochondrial *COX3* gene using mit1C primers and an exogenous internal amplification control as previously described [30].

### 2.4. Targeted Amplicon Sequencing

TAS was performed using the ChapterDX *Cyclospora* Target Enrichment NGS Assay kit (Chapter Diagnostics, Menlo Park, CA, USA) according to the manufacturer’s protocol, as detailed previously [24], with a 35 min bait-capture hybridization step for clinical specimens and 60 min for inoculated raspberry samples. In brief, target-specific multiplex PCR for 52 genomic loci was performed using 5 µL of DNA, without adjusting for the concentration after extraction, followed by a hybridization capture step in which DNA baits with target sequences were used. Subsequently, a second PCR was utilized for the addition of barcodes and adapters for use with Illumina sequencing platforms (Illumina, San Diego, CA, USA). A subset of 12 clinical specimens were also used in a TAS assay without baits using the ChapterDX *Cyclospora* NGS kit (Chapter Diagnostics), following the manufacturer’s protocol. This kit, which targets the identical 52 loci as the kit that includes the hybridization capture step, employs a rapid and straightforward one-step PCR setup, followed by a purification step, resulting directly in libraries for sequencing on an Illumina platform. For each sample, 5 µL DNA was used in a single multiplex PCR containing both target-specific primers and primers for the addition of barcodes and adapters, followed by a SPRIselect bead (Beckman Coulter, Brea, CA, USA) purification step. Along with using the PCR protocol included with the kit for the 12 specimens, the same 12 specimens were subjected to a touchdown PCR protocol substituted for the PCR protocol supplied. The touchdown PCR protocol included five cycles at each annealing temperature of 65 °C, 62 °C, and 60 °C (15 cycles total) in place of the 10 cycles at 60 °C specified in the kit instructions. Sequencing libraries were quantitated using the Qubit High-Sensitivity Assay (Qubit, London, UK) and inspected for quality using TapeStation 4150 (Agilent, Santa Clara, CA, USA). The TAS libraries were sequenced on an Illumina MiSeq platform generating paired-end 249 bp reads. Twelve clinical specimens were pooled in a single sequencing run, while six inoculated raspberry samples were pooled per run. Sterile water and uninoculated raspberries yielded libraries too dilute for sequencing and without expected library fragment sizes.

### 2.5. Multi-Locus Sequencing Typing

While the TAS panel and the eight-marker MLST panel largely overlap, due to challenges in designing primers that avoid undesirable primer interactions (e.g., primer dimers), parts of certain markers in the MSLT panel were not completely covered by the TAS marker panel [24]. To fill sequence gaps in the data obtained from the TAS assay compared to the eight-loci method, thus allowing a direct comparison between both sets of markers, the HC360i2, MSR, and Mt-junction markers were sequenced using the primers specified in the CDC MLST method [18] but with a modified PCR protocol. The DNA extracted from the clinical specimens was purified using the QIAquick PCR Purification Kit (Qiagen, Germantown, MD, USA). For all three markers, 25 µL PCR reactions consisted of 22 µL of Platinum PCR SuperMix High-Fidelity Master Mix (Invitrogen, Carlsbad, CA, USA), 1 µL each of the forward and reverse primers at a 200 nM final concentration, and 1 µL of DNA template. The PCR conditions were 94 °C for 2 min, followed by 40 cycles of 94 °C for 15 s, 55 °C for 30 s, and 68 °C for 40 s, with a subsequent final extension cycle of 68 °C for 8 min. The PCR products were visualized using a TapeStation 4150 and then purified using SPRIselect beads with a 1.8X ratio and yielding 18 µL purified amplicons in low TE buffer (G-Biosciences, St. Louis, MO, USA). Purified amplicon concentrations were determined using the Qubit High-Sensitivity Assay, and the amplicons were pooled in equal concentrations for each clinical specimen. Sequencing libraries were prepared using the DNA Prep Kit (Illumina) and sequenced on an Illumina MiSeq sequencer, resulting in paired-end 250 bp reads.

### 2.6. Bioinformatic Analyses

Quality control was performed on fastq files using FastQC (http://www.bioinformatics.babraham.ac.uk/projects/fastqc, accessed on 10 January 2023). Sequence reads were processed, and haplotyping was performed as previously described [24]. Haplotypes were determined to be present if there were at least 10 matching reads and the matching reads constituted at least 10% of the total reads for the marker. The number of reads in the datasets for each sample matching the *C. cayetanensis* genome sequence and matching other genera in the family *Eimeriidae* was determined as detailed earlier [24]. The Eukaryotyping program (R scripts) from the CDC [15] uses a heuristic algorithm to generate a pairwise distance matrix of the samples from the haplotype presence/absence data. This R script algorithm was translated to a custom Python program, heuristic_CHDX.py (github.com/mmammel8/partitioning, accessed on 10 October 2023), and modified so that it did not impute average values for missing amplicon data but instead used pairwise deletion where data were missing, scaling the computed distance to the full set of 52 amplicons. For the distance matrix based on the eight markers in the CDC MLST scheme, the results from the MLST sequencing for markers HC360i2, MSR, and Mt-junction were used to fill gaps in the TAS assay results as needed to obtain haplotype results based on the full length of all eight MLST markers. A dendrogram was also constructed to include genotypes extracted from *C. cayetanensis* genome assemblies that are publicly available in NCBI. For this, haplotypes were assigned for the assemblies as previously described [24]. Pairwise distance matrices were generated from the haplotype presence/absence results using the custom program employing pairwise deletion to manage missing data for the respective genotyping panel for all cluster dendrograms. The distance matrices were used for generating dendrograms using hierarchical clustering performed in R using Agnes [31] as described previously [24].

Several methods were used to determine the optimal partition number for the hierarchical genetic cluster dendrograms. First, a program for hierarchical tree dissection [26] was modified and utilized. The program as downloaded (https://github.com/Joel-Barratt/Hierarchical-tree-dissection-framework, accessed on 11 September 2023) [27] determines a cutoff height separately for the two main branches expected to contain the two lineages A and B as defined by the CDC [4]. For this dataset, there was not a complete separation of the two proposed genetic lineages into two main branches; thus, to yield consistent agnostic cluster partitioning among all specimens, we modified the program to treat all strains similarly. The modified custom Python program, clustering4-dx.py, is available on GitHub (github.com/mmammel8/partitioning, accessed on 10 October 2023). In brief, from the heuristic distance matrix, hierarchical clustering using the Ward method was performed using the SciPy linkage function. Using the SciPy cut_tree function, the maximum number of clusters that would include at least two of the samples in each cluster was determined. Over 1000 iterations, two strains were randomly selected from each cluster, all distances of selected strains were sorted, and the distance at the 5th percentile was stored. The average of the 5th percentile distances for the 1000 iterations was used as the threshold for determining the minimum number of clusters in which 99.5% of the intracluster distances were below the threshold. Statistics for three other methods for investigating the optimum number of partitions to select for genetic clustering were computed from the distance matrix using the R package fpc v. 2.2-10 (https://CRAN.R-project.org/package=fpc, accessed on 11 September 2023), function cluster.stats. These methods included the elbow method (within.cluster.ss) [32], average silhouette width (avg.silwidth) [33], and the Dunn index (dunn) [34]. Two additional tests, TreeCluster [35] and PARNAS [36], were used to investigate partitioning of genetic clusters, both using a hierarchical tree based on the distance matrix. TreeCluster was run with the max_clade option and supplied with the 5th percentile distance determined previously for the threshold. PARNAS was run with the cover option to specify the radius, where the 5th percentile distance was used.

## 3. Results

### 3.1. Genotyping Clinical Specimens

To evaluate the performance of the TAS assay for genotyping clinical specimens and the genetic resolving power obtained, 66 clinical specimens (Table 1) were genotyped with the hybridization capture step included (Table 2). Twelve sequencing libraries were pooled in a sequencing run generating from 2.33 to 5.33 million reads per sample (average 3.15 million reads). The percentage of reads matching to the *C. cayetanensis* genome for each sample ranged from 72.6 to 98.2% (average 90.1%), and all datasets contained ≤8 reads matching other genera in the *Eimeriidae* family. All 52 TAS markers were successfully haplotyped for 14 of the specimens, and for all specimens, ≥50 markers were haplotyped. For markers that could not be haplotyped, either there were no matching reads in the sequence dataset or the marker did not pass the read depth criteria. Marker CH in the TAS assay [24] amplifies the same region as the mitochondrial junction variable repeat region marker (Mt-junction) in the eight-marker MLST genotyping scheme [18]. For 49 of the specimens, all markers were haplotyped except marker CH, which does not have a corresponding bait in the TAS with the enrichment kit. Specimen CDC56 also had 51 markers haplotyped, but in this case, the missing marker was marker AK, which contains eight SNP sites; thus, a total of 388 SNP sites were included in the analysis for this specimen. For CDC44 and CDC50, markers CH and FA were not haplotyped, resulting in inclusion of a total of 380 SNP sites in the analysis. The number of different haplotypes discovered for each of the 52 markers among the 66 specimens ranged from 2 to 10, with 3 being most frequent (Table S1). The five markers with only two haplotypes represented among all specimens were AA, CA, CB, CC, and CD. CA, CB, CC, and CD correspond to the CDS1, CDS2, CDS3, and CDS4 markers, respectively, in the eight-marker MLST genotyping scheme. If a specimen contains a single strain of *C. cayetanensis*, it would be expected to have no more than one or two haplotypes per marker for markers targeting the nuclear genome. Inspection of the haplotype results revealed four specimens for which at least one nuclear marker had more than two haplotypes identified. These specimens, CDC34, CDC58, CDC60, and CDC64, had five, two, three, and two markers, respectively, with greater than two haplotypes.

### 3.2. Genotyping without Hybridization Capture Enrichment

Since the parasite load in most clinical specimens is higher than in food and environmental samples, we assessed the need for the extra enrichment step for sequence library preparation. Preparation of sequence libraries for genotyping specimens containing *C. cayetanensis* using the same 52 markers, but without the bait-capture step, can be accomplished in a simplified one-step PCR, followed by purification. We evaluated the version of the TAS assay without the bait-capture enrichment step on a subset of 12 fecal specimens for comparison (Table 2). There was a decrease in the percentage reads matching the *C. cayetanensis* genome sequence without the use of the bait-capture step (average 14.1%) compared to that with the baits (average 87.5%). However, the number of markers haplotyped was similar with the exception of one specimen, CDC46, for which only 47 markers could be haplotyped based on read depth criteria. CDC46 had the lowest percentage of reads matching *C. cayetanensis* amplicon sequence and likely the lowest parasite load. For all specimens with less than 52 markers haplotyped, marker AK was not haplotyped. The DNA size profiles in the sequencing libraries prepared with the TAS assay kit without the bait-capture step revealed the presence of off-target amplification. In an attempt to increase on-target amplification when using the kit, the target-specific PCR protocol was modified to contain a touchdown PCR method. While the touchdown PCR protocol resulted in a lower percentage of reads matching *C. cayetanensis* for specimen CDC04, the percentage was increased in all other specimens (Table 2). Marker AK could not be haplotyped due to lack of sequence read coverage in any of the 12 specimens when using touchdown PCR; however, an additional three markers were haplotyped for CDC46, containing a total of 19 SNPs. Although a bias in sequencing depth for specific markers was observed when comparing results for the two PCR protocols, the difference was less than one percent for all but four markers, and even for those four markers, it was never greater than six percent.

### 3.3. Genetic Cluster Resolution

**Figure 1:**
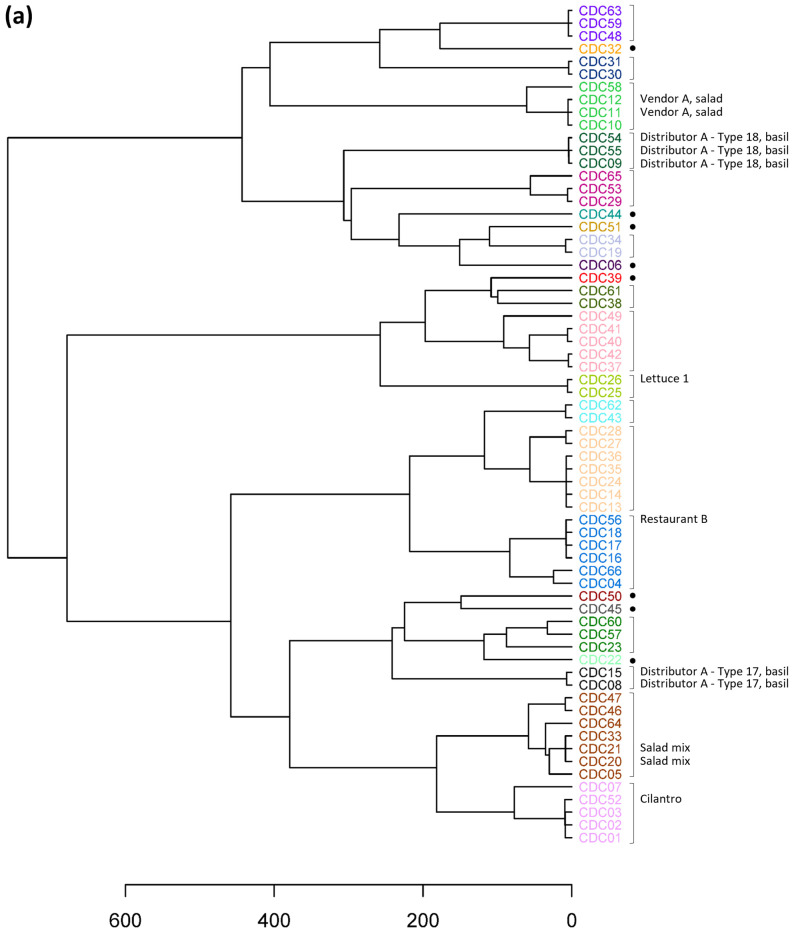

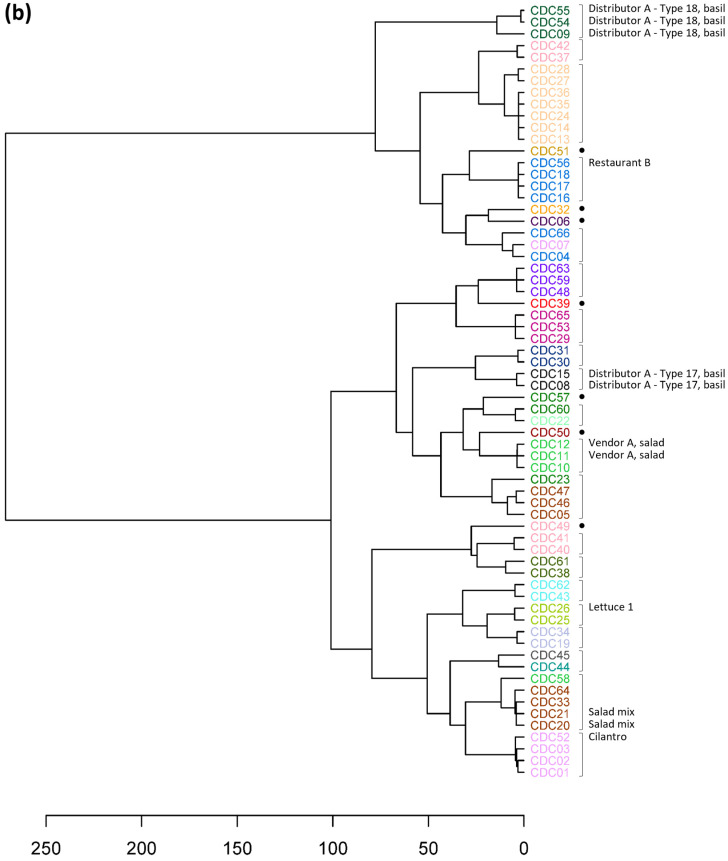
Hierarchical cluster dendrograms for clinical specimens based on haplotype results using (**a**) the 52-loci targeted amplicon sequencing (TAS) panel and (**b**) the 8-loci MLST panel. The 24 clusters resulting from partitioning the TAS dendrogram are denoted by the specimen label font color, along with either a bracket or a dot for clusters containing a single specimen. The 27 clusters resulting from partitioning the MLST dendrogram are denoted by brackets or dots. The dendrogram was dissected using the modified version for partitioning stated in this work that was based on a previously described method [26].

A dendrogram was generated from a distance matrix computed based on TAS panel haplotype results for the 66 clinical specimens (Figure 1a). To dissect the hierarchical tree into biologically relevant discrete partitions, where specimens within a cluster have a high probability of containing the same *C. cayetanensis* strain, we used a modified program based on the method used by the CDC that was found to yield epidemiologically meaningful results [26]. The minimum number of clusters in which 99.5% of the intracluster distances were below the computed threshold of 109.0 was determined to be 24. For comparison, other methods of partitioning the dendrogram were explored. The number of optimum clusters determined using the silhouette and Dunn index methods were 23 and 38, respectively, with an average silhouette width of 0.620 and a Dunn index of 2.71. The elbow method yielded a smooth curve; thus, a partition number was not obtained. TreeCluster and PARNAS both produced an optimum partition number of 23. To evaluate differences in genetic resolution and clustering of the specimens between the 52-loci TAS panel used in this study and the 8-loci MLST panel currently in use [18], the haplotype results for the subset of markers representing the eight MLST markers were extracted from the TAS results. The TAS assay does not completely cover the full length of the HC360i2 MLST marker but does include 20 of the 24 SNP sites in the marker [24]. Although all five of the known SNP sites in MLST marker MSR are included within two markers in the TAS panel overlapping MSR, there is a 160 bp stretch of sequence in MSR not included in the TAS panel sequence. Additionally, the Mt-junction marker was not haplotyped in 49 of the 66 specimens included in this study. As a result, for those three markers, the missing sequence data were supplied by utilizing traditional PCR with the MLST primers and approach. With this strategy, haplotype results for all eight markers in the MLST scheme were determined for all specimens except for the Mt-junction marker in specimens CDC11 and CDC26, for which PCR product was not obtained. A distance matrix was calculated based on the haplotype results for the eight markers and was used to create a hierarchical tree and determine optimal cluster partitioning, as was performed for the TAS assay (Figure 1b). For the eight-loci MLST scheme, the threshold was calculated to be 15.54, resulting in 27 clusters. The optimum number of clusters based on the silhouette, Dunn index, TreeCluster, and PARNAS methods were 26, 35, 28, and 28, respectively, with an average silhouette width of 0.561 and a Dunn index of 1.13.

The increase in genetic cluster resolution gained from the inclusion of additional phylogenetically informative genomic content for genotyping can be observed in the dendrogram branch heights and results, in some cases, in substantial differences in the specimens included in individual clusters (Figure 1). Rather than the division of clusters in the TAS dendrogram into neighboring but smaller clusters in the MLST dendrogram, there is movement of clusters and individual specimens into or out of clusters. For example, specimens CDC37, CDC42, CDC40, CDC41, and CDC49 comprise a cluster in the TAS dendrogram that is broken into three clusters in the MLST dendrogram, one of which, including CDC37 and CDC42, is in a very different location with different neighboring clusters. In fact, the specimen composition was identical between the two dendrograms for only 15 clusters. While there were eight and seven clusters containing single specimens in the TAS and MLST dendrograms, respectively, only five of the specimens are in common. More detailed inspection of the movement of specimens in terms of the number of markers with different haplotypes provides perspective. Although CDC44 and CDC45 differ in haplotype for two of the eight MLST markers (CDS1 and MSR) and cluster together in the MLST dendrogram (Figure 1b), they differ in 33 of the 41 new markers in the TAS assay and are distant from each other in the TAS dendrogram (Figure 1a). In another case, CDC22 and CDC60 have identical haplotypes in the eight-loci MLST scheme, with the exception of a second haplotype for markers CDS2 and HC360i2 in CDC22 not found in CDC60, and these specimens cluster together. However, a second haplotype was observed in the CDC60 results that was not found in CDC22 for 20 of the new markers, the haplotype results differed completely for 8 of the new markers, and the specimens were placed in separate clusters in the TAS dendrogram. In another example, in the MLST scheme, CDC07 and CDC04 differ only in haplotype for marker CDS2 and are in the same cluster (Figure 1b), while they differ in haplotype for an additional 15 of the new markers in the TAS panel and are located in different clusters in the dendrogram (Figure 1a).

### 3.4. Application of Epidemiologic Information

After partitioning the hierarchical tree into the optimal number of genetic clusters, the available corresponding metadata were used to consider the epidemiologic relevance of the resulting clusters (Table 1, Figure 1a). The specimens were collected during years 2018–2022, and while most had unknown epidemiologic linkage, 12 specimens had associated epidemiologic information. In all cases, specimens with the same epidemiologic source information were assigned to the same cluster, and those clusters were separate from clusters containing specimens with different source information. It may be possible to infer the source associated with specimens lacking epidemiologic information if a specimen within the cluster has known source linkage and a similar collection date (Table 1, Figure 1a). For example, CDC01, CDC02, CDC03, CDC07, and CDC52 belong to the same genetic cluster; however, only CDC52 had a known epidemiologic linkage. Further examination showed that all samples within this cluster were collected between 10 August and 26 August 2020 (Table 1). Additionally, all samples in this cluster were sent to CDC from public health labs in the Southeastern United States, which suggests these samples may all be related to the same exposure, even though only one was part of the identified epidemiologic cluster. Other specimens with unknown epidemiologic association also share genetic relatedness with specimens linked to sources, namely prepackaged salad mix and Restaurant B. For Restaurant B, CDC56 was the only epidemiologically linked specimen in its cluster, yet three other specimens in that cluster were collected within 3 weeks of CDC56 in July 2019, and they are all from Massachusetts, which supports a close relationship. CDC10 may share the same source as CDC11 and CDC12, namely Vendor A, a fresh produce vendor epidemiologically linked to a large outbreak of cyclosporiasis in 2018 [18]. This claim is supported by the fact that these three samples originate from the same state, Iowa, and year. Interestingly, CDC58, collected four years later, is also genetically related to those three specimens collected in 2018. The other three specimens in the cluster have a tighter genetic relationship, suggesting that if CDC58 is the same strain, there might have been some genetic changes over time. In fact, seven of the 24 clusters are composed of specimens that were collected in different years. It is also noteworthy that with the added markers in the TAS panel, CDC58 markedly changed position within the tree. This specimen clusters alongside specimens associated with Vendor A in the hierarchical tree computed using the 52-loci TAS marker panel (Figure 1a), while it clusters more closely with specimens linked to prepackaged salad mix when the 8-loci MLST marker panel is employed (Figure 1b), noting that specimen CDC58 is not identical to specimens from either outbreak. This highlights the benefit afforded by sequencing a greater number of markers, where specimens initially perceived as being genetically similar to one another become more disparate as additional parts of the genome are sampled.

Along with identifying specimens that may share a common source, we sought to determine whether some specimens are linked genetically to specimens from countries outside the USA. To this end, a hierarchical cluster dendrogram was generated from a distance matrix computed using haplotype results from both the set of 66 clinical specimens included in this study and 40 WGS assemblies available at NCBI (Figure S1). Applying the same method for determining the optimal number of partitions as was used for the TAS and MLST dendrograms (Figure 1), the TAS plus WGS dendrogram was divided into 31 clusters (Figure S1). There were three clusters that contain both a WGS strain from a country outside the USA and specimens included in this study. CDC45, a specimen collected in 2022 with unknown epidemiologic linkage, clustered with a strain from Indonesia (accession JAHWDO01). All seven specimens clustering with CDC21 in the TAS dendrogram (Figure 1a) were placed with a Canadian strain (accession JAHEWR01) (Figure S1). Finally, nine specimens with collection dates including 2020, 2021, and 2023 clustered with a WGS strain from Mexico (accession RDRN01). The cluster was also composed of WGS strains from Texas. In a different cluster, WGS strains from Texas clustered with CDC52, the specimen associated with cilantro by epidemiology.

### 3.5. Assessment of Traceback Utility

**Figure 2:**
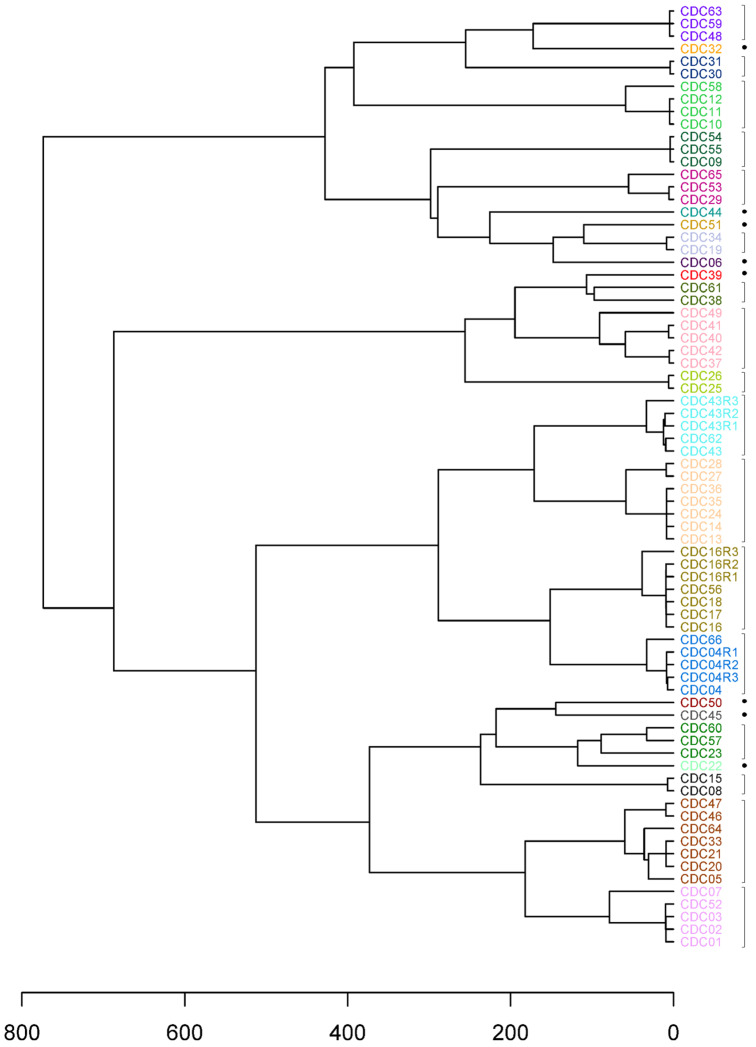
Hierarchical cluster dendrogram based on haplotype results from both clinical specimens and raspberries inoculated with clinical specimens. The raspberry sample names correspond with the dilutions listed in Table 3. The 25 clusters determined by partitioning the dendrogram are denoted by sample label font color, along with brackets or dots. The dendrogram was dissected using the modified version for partitioning stated in this work that was based on a previously described method [26].

The ability to perform traceback analysis by genetically linking a contaminated food product with clinical specimens was assessed using DNA prepared from raspberry samples inoculated with fecal specimens. Three of the clinical specimens, CDC04, CDC16, and CDC43, were used, along with two different dilutions of the fecal specimens (Table 3). RT-PCR for the detection of *C. cayetanensis* was performed on DNA extracted from the fecal specimens, as well as DNA from the nine inoculated raspberry samples. As expected, the C_T_ values increased with increasingly dilute fecal inoculum (Table 3). Genotyping was performed on the nine inoculated raspberry samples using the TAS kit, including the bait hybridization step for enrichment. The number of reads per sample ranged from 3.56 to 6.54 million (average 5.06 million), and the percentage of reads matching to the *C. cayetanensis* genome averaged 69.3%, lower than the average for datasets of the 66 clinical specimens using the kit with the baits but much higher than for the clinical specimens without the baits (Table 2). At least 50 of the 52 markers were haplotyped for all inoculated raspberry samples with the exception of CDC04R3 and CDC16R3, two of the samples receiving the most dilute fecal inoculum. Of note, CDC04R3 and CDC16R3 had the highest C_T_ values in the RT-PCR detection assay (Table 2). The sequence dataset for CDC04R3 contained sequence matching 31 markers, but only 29 markers were haplotyped due to a lack of read depth for 2 markers. Similarly, sequence matching 25 markers was obtained for CDC16R3, but only 21 markers had sufficient read depth to pass the minimum cutoff for haplotyping. A hierarchical cluster dendrogram was generated from the distance matrix computed based on haplotype results from the 66 clinical specimens, along with the 9 inoculated raspberry samples (Figure 2). Calculation of the optimal number of partitions for the dendrogram resulted in 25 clusters (threshold 101.7), an increase of 1 cluster compared to the dendrogram that did not include the contaminated raspberry samples. The composition of the clusters remained unchanged for all clusters except the cluster comprising CDC04, CDC16, and four additional specimens. This cluster split into two clusters, one containing CDC04 and the three raspberry samples inoculated with CDC04 as well as specimen CDC66. The other cluster was composed of CDC16, along with three raspberry samples inoculated with CDC16 and three other specimens (Figure 2). For comparison, a dendrogram was created using a distance matrix in which distances between markers with missing haplotype results were computed using the original method in the CDC program [15] rather than the pairwise deletion method utilized in this study. The resulting dendrogram was similar to the dendrogram computed using the pairwise deletion method (Figure 2), with the exception that CDC16R3 and CDC04R3 were each placed in their own individual cluster.

## 4. Discussion

We recently reported the development of a TAS method for genotyping *C. cayetanensis* from fresh produce and clinical samples with enhanced genomic resolution and sensitivity [24] compared to the eight-loci MLST method currently used by the CDC [14]. Along with genotyping fresh produce items inoculated with low levels of *C. cayetanensis* oocysts, the utility of the TAS method for genotyping from several challenging clinical specimens with low parasite loads was previously demonstrated [24]. In the present study, to further assess the performance of the assay, we genotyped 66 genetically diverse fecal samples from cyclosporiasis cases. Our genotyping results utilizing the TAS kit without the extra hybridization step on a subset of 12 specimens in this study suggest that, in general, the bait-capture step is not necessary for fecal specimens. However, as noted in the previous study [24], the use of the extra enrichment step on some specimens with low parasite loads or specimens that might not have been stored ideally would increase the number of markers haplotyped, leading to a possible practical strategy of genotyping without the baits, followed by use of the kit with the baits, to repeat any specimens with too low a number of markers haplotyped initially. It has been reported that when utilizing the eight-marker MLST method, there were specimens with markers that could not be haplotyped due to PCR amplification failure, resulting in failure rates for passing the genotyping criteria of 21% [18] and 19% [21] of the specimens. It is worth noting that with the TAS kit, ≥50 of the 52 markers were haplotyped for all 66 specimens, thus demonstrating the greater robustness of the genotyping method. Touchdown PCR is often employed for increasing the specificity and sensitivity of standard PCR protocols [37,38]. Our attempt to increase the on-target sequencing by modifying the PCR program to consist of a touchdown approach was moderately successful in that it did increase the percentage of reads matching *C. cayetanensis* sequence, but not nearly to the levels obtained by including the bait-capture step. Although the substitution of a touchdown PCR protocol can bias the relative abundance of the different marker amplicons, resulting in too low an abundance of some amplicons for haplotyping, our results demonstrated very minimal impact on marker sequence depth for the touchdown PCR protocol applied in this study. Clearly, our results demonstrate that utilizing the baits would allow for multiplexing a greater number of specimens per sequencing run, a factor to consider when determining cost effectiveness. Nevertheless, if using the TAS method without the bait-capture step, applying the touchdown PCR method could prove advantageous.

The haplotype results from the 66 specimens included in this study can be used to evaluate the genomic loci included in the TAS assay marker panel for the purpose of modifying the panel. The cluster assignments in the dendrogram depend on the combined haplotype distances for all markers. For each marker, there is a distribution of haplotypes among the strains, and most markers produce different distributions among the specimens. As the number of different distributions is increased, the accuracy of the genetic clustering increases. The fact that only two different haplotypes were observed for markers AA, CA, CB, CC, and CD suggests that these markers are less useful for genetic discrimination between strains and should be considered for replacement in future TAS marker panel designs. In agreement, replacing CDS1, CDS2, and CDS4 in the eight-loci scheme (CA, CB, and CD in the TAS assay) has previously been advised based on low Shannon entropy and the inability to haplotype the markers in many specimens [21]. The change we observed in the composition of the clusters between the two genotyping schemes demonstrate that the eight-loci scheme is not representative of the *C. cayetanensis* genome, an expected result given the substantial difference in number of informative loci between the panels. This is highlighted by our analysis of the number of markers in the two genotyping schemes with observed haplotype differences between specimens for several examples and the different placement of those specimens in the dendrograms. With this in mind, ideally, along with replacement of certain markers, the identification of additional informative loci to add to the panel would be advisable as long as sensitivity is not compromised. The recognition of the need to expand the number of markers beyond the eight-loci scheme previously identified has led to recent work analyzing both the nuclear and mitochondrial genome for potential subtyping loci [39]. Although 47 potential markers were identified, the entropy of the loci should be computed to determine the utility before consideration for inclusion in a genotyping panel.

Genotype comparison among cyclosporiasis specimens is complex due to the possible genetic heterozygosity of the parasite at the loci in the nuclear genome, as well as the possibility of mixed strains within a specimen. In fact, we identified four specimens that appear to contain more than one *C. cayetanensis* strain. This complexity also leads to greater difficulty in determining the appropriate cutoff height to use for partitioning a hierarchical tree into meaningful clusters, an important consideration in support of epidemiologic investigations, as the partitioning impacts the interpretation of the genetic relatedness of the specimens. Several methods for determining the optimal number of clusters in the genetic distance dendrograms were explored in this study and demonstrate the difference in the resulting genetic resolution when utilizing the TAS assay with 52 markers in comparison to the MLST method with eight markers. The higher Dunn Index value, the ratio of the minimum intercluster distance, and the maximum intracluster distances, obtained with the TAS assay, indicates a more optimal clustering solution with greater distinction between clusters. Similarly, the higher silhouette width, an indication of how well each specimen matches within the cluster and poorly to other clusters, obtained with the TAS assay reveals a gain in genetic resolution. We chose to use the method described previously for computing a threshold value for dissecting the hierarchical trees based on the fact that it was demonstrated to produce epidemiologically relevant clusters using a large set of specimens [26]. Importantly, it was noted in studies describing early iterations of the tree dissection method used here that the method is best suited to larger datasets than those analyzed in the present work; thus, direct comparisons of the TAS and eight-marker methods based on the way in which the trees are dissected should be viewed with some caution. Nevertheless, sets of clusters were identified in an unbiased way, and the partitions identified include samples possessing comparatively close genetic relationships. The silhouette, PARNAS, and TreeCluster methods resulted in the same number of partitions and are in close agreement with the empiric 5th percentile method, while the optimum number of clusters using the Dunn Index was much higher. However, for PARNAS and TreeCluster, the user-specified parameters were set at values established for the empiric 5th percentile method, thus, were not computed completely independently of that method. In contrast, in a previous work utilizing the eight-loci genotyping scheme on a large and diverse set of specimens, the silhouette method assigned specimens to far fewer partitions that were not considered epidemiologically meaningful compared to the empiric 5th percentile method [26]. The number of genetic clusters reported based on haplotype results from cyclosporiasis specimens using the eight-loci MLST scheme has increased from 21 [18,21] to 46 [26] with the addition of new genotypes to the CDC reference database over the past two years. Our result of 27 clusters using the eight-loci scheme reveals that the 66 specimens included in this study are an under-representation of known overall genetic diversity; therefore, we would expect greater than 24 clusters if using the TAS scheme on a larger number of the CDC reference set of specimens. However, the decrease in the number of optimum clusters from 27 to 24 with the increase in informative genomic loci targeted from 8 to 52 for the set of 66 specimens implies that the 8 loci used in the MLST scheme over-represent the genetic diversity among strains.

The results from this study cannot be used to fully evaluate the performance of the TAS and dissection of the hierarchical tree into an appropriate number of genetic clusters due to these limitations: the study includes a comparatively small dataset, and the number of specimens with associated epidemiologic data is limited. A larger number of specimens possessing epidemiologic links would allow us to better demonstrate the ability of the TAS panels to distinguish outbreaks caused by related, yet different, strains compared to the eight-marker MLST panel. However, we were able to demonstrate that within the set of specimens included, cluster assignments were consistent with the known epidemiologic data, even though the composition of some clusters changed compared to those determined using only the original eight MLST markers. The increased confidence in the placement of specimens within clusters when utilizing the TAS genotyping panel will aid in more accurate source tracking. Imported fresh produce has been implicated in many outbreaks in the USA, and travel-related cases of cyclosporiasis have been reported [8,40]. Genetic linkages of specimens collected inside the USA to those collected outside the USA, as were demonstrated in this study, may prove valuable in determining travel-related cases of cyclosporiasis, as well as any association with imported fresh produce and the geographic scope of outbreaks in the future. For example, our genetic linkage of specimens associated with prepackaged salad mix and a specimen collected in Canada is noteworthy. Travel-related information for the 66 specimens in the current study is unknown; therefore, we cannot confirm the cluster assignment results arising from travel. As additional specimens are added to a genetic distance analysis, cluster assignments of specimens may change, as was observed using the eight-loci MLST method for genotyping [22] during a cyclosporiasis outbreak in 2020 linked by epidemiology to bagged salad mix. It is expected that as the number of phylogenetically informative markers is increased, the genetic cluster stability will be improved. Future work involving genotyping a larger set of specimens would be necessary to demonstrate whether the TAS marker panel leads to greater cluster stability. Additionally, future studies evaluating the TAS method should include a greater number of samples that have been linked epidemiologically to outbreak clusters of cyclosporiasis.

Ideally, a *C. cayetanensis* genotyping method would be used not only for discovering genetic linkages between clinical specimens but also for linking clinical specimens to contaminated food items. Multiple cyclosporiasis outbreaks have been epidemiologically linked to contaminated fresh berries, and *C. cayetanensis* has been detected on berries in surveillance studies [41,42,43,44,45]. In our previous study, we demonstrated the ability to genotype and accurately cluster a blackberry sample inoculated with 10 purified *C. cayetanensis* oocysts [24]. The results in this current study expand on genotyping experiments utilizing this important food matrix by directly inoculating clinical specimens rather than purified oocysts onto raspberry samples. A comparison of the C_T_ values for the low inoculum samples CDC04R3 and CDC16R3 with those obtained from fresh produce inoculated with purified oocysts [24] suggests that those two raspberry samples contain ≤10 oocysts within the fecal inoculum, while the other inoculated raspberry samples likely contain >100 oocysts. Raspberry samples were deliberately inoculated with dilute fecal specimen to replicate the possibility that naturally contaminated samples might yield a low number of markers for genotyping. It is important for the genotyping and clustering methods to be robust enough to link food samples with clinical specimens, even in the absence of haplotype results for some markers. Significantly, although the original cluster containing CDC04 and CDC16 was divided into two partitions, the inoculated raspberry samples clustered with the respective clinical specimens despite the lower number of markers haplotyped for the most dilute inoculum samples. However, due to the division of the cluster, certain specimens that occupied the same cluster in the dendrogram without inclusion of the inoculated raspberry samples would now be considered less genetically related. The method employed in this study for computing the distance matrix by using a pairwise deletion method to handle missing data was a considerable improvement over the previous method [15], as exemplified by the fact that the most dilute inoculum samples were placed in clusters with the respective fecal specimen. The inclusion of a greater number of clinical specimens in the analysis would also be expected to improve the accuracy and emphasize the utility of including as many specimens in a reference database as possible. Nevertheless, the development of bioinformatic methods to enhance the accuracy of phylogenetic relatedness among *C. cayetanensis* strains is a critical future area to explore. Our results also suggest that any modifications that can be made to the methods used to obtain the microbiome from food samples yielding a higher ratio of *C. cayetanensis* DNA to microbial background DNA are worth investigating, as it would lead to improved source tracking abilities, with a greater number of markers haplotyped for very low contamination samples.

## 5. Conclusions

Taken together, our genotyping and hierarchical clustering results provide valuable insights into how the inclusion of many more phylogenetically relevant nuclear loci in a targeted amplicon genotyping scheme for *C. cayetanensis* affects the computed genetic relatedness of clinical specimens. The gain in genomic resolution demonstrated in this study by using the TAS assay for genotyping compared to the current eight-loci MLST scheme will be beneficial for source tracking and determining the inclusion/exclusion of cyclosporiasis cases in outbreaks, as well as for exploring *C. cayetanensis* genome diversity and temporal variation worldwide. Furthermore, our results demonstrating the ability to correctly place raspberry samples inoculated with diluted fecal specimens in a hierarchical cluster dendrogram indicate that the TAS assay will be useful for genetically linking contaminated food items with clinical specimens, and also highlight the need for further bioinformatic work related to genotyping and meaningful genetic clustering of this important foodborne parasite. The success obtained in genotyping both food items contaminated with a very low number of *C. cayetanensis* oocysts and clinical specimens has prompted testing of additional loci for genotyping and led to an improved design of the TAS assay marker panel. The new version is currently undergoing testing on clinical and food samples.

## Figures and Tables

**Table 1:** Clinical specimens used in this study and associated epidemiologic information.

Sample	C_T_	Collection Date	Epidemiologic Information
CDC01	25.83	17 August 2020	Unknown
CDC02	24.89	18 August 2020	Unknown
CDC03	27.20	10 August 2020	Unknown
CDC04	26.05	6 August 2020	Unknown
CDC05	24.47	21 July 2020	Unknown
CDC06	N/A	1 September 2020	Unknown
CDC07	19.58	26 August 2020	Unknown
CDC08	21.62	26 June 2019	Distributor A—Type 17, basil
CDC09	25.91	2 July 2019	Distributor A—Type 18, basil
CDC10	N/A	2018	Unknown
CDC11	N/A	2018	Vendor A, salad
CDC12	N/A	2018	Vendor A, salad
CDC13	21.33	Unknown	Unknown
CDC14	30.51	Unknown	Unknown
CDC15	23.24	31 July 2019	Distributor A—Type 17, basil
CDC16	23.46	25 July 2019	Unknown
CDC17	21.96	19 July 2019	Unknown
CDC18	22.79	19 July 2019	Unknown
CDC19	21.65	13 July 2019	Unknown
CDC20	30.86	26 June 2020	Prepackaged salad mix
CDC21	30.42	29 June 2020	Prepackaged salad mix
CDC22	25.66	Unknown	Unknown
CDC23	23.62	20 July 2020	Unknown
CDC24	22.61	Unknown	Unknown
CDC25	22.86	15 July 2021	Unknown
CDC26	25.42	9 July 2021	Lettuce 1
CDC27	23.75	13 July 2021	Unknown
CDC28	20.48	13 July 2021	Unknown
CDC29	27.60	30 July 2021	Unknown
CDC30	20.58	6 July 2019	Unknown
CDC31	N/A	2018	Unknown
CDC32	19.99	12 August 2021	Unknown
CDC33	23.50	29 July 2022	Unknown
CDC34	27.56	29 July 2022	Unknown
CDC35	19.34	29 July 2022	Unknown
CDC36	23.93	19 July 2022	Unknown
CDC37	20.77	10 August 2022	Unknown
CDC38	22.76	14 June 2022	Unknown
CDC39	21.49	20 July 2022	Unknown
CDC40	20.31	2022	Unknown
CDC41	25.16	21 July 2022	Unknown
CDC42	22.94	13 July 2022	Unknown
CDC43	19.78	3 August 2022	Unknown
CDC44	23.68	24 June 2022	Unknown
CDC45	25.46	8 August 2022	Unknown
CDC46	27.03	15 July 2022	Unknown
CDC47	21.91	2022	Unknown
CDC48	21.61	5 July 2022	Unknown
CDC49	22.23	8 August 2022	Unknown
CDC50	27.96	12 April 2022	Unknown
CDC51	21.29	12 August 2020	Unknown
CDC52	28.25	21 August 2020	TN/GA/VA Mexican-style restaurant, cilantro
CDC53	23.94	9 July 2021	Unknown
CDC54	26.09	16 July 2019	Distributor A—Type 18, basil
CDC55	25.03	9 July 2019	Distributor A—Type 18, basil
CDC56	26.46	3 July 2019	Restaurant B, Romaine lettuce or basil
CDC57	22.09	25 June 2022	Unknown
CDC58	28.06	23 July 2022	Unknown
CDC59	26.53	5 July 2022	Unknown
CDC60	28.38	27 June 2022	Unknown
CDC61	23.74	1 July 2022	Unknown
CDC62	27.62	31 July 2022	Unknown
CDC63	26.93	20 July 2022	Unknown
CDC64	31.76	14 August 2022	Unknown
CDC65	18.17	19 July 2022	Unknown
CDC66	23.51	8 August 2022	Unknown

**Table 2:** Comparison of targeted amplicon sequencing results with and without the use of baits for the enrichment of a target-specific PCR product.

	Baits			No Baits			No Baits, Touchdown PCR		
Sample	%Ccay ^1^	Markers ^2^	SNPs ^2^	%Ccay ^1^	Markers ^2^	SNPs ^2^	%Ccay ^1^	Markers ^2^	SNPs ^2^
CDC04	80.7%	51	396	32.0%	52	396	23.0%	51	388
CDC07	81.3%	52	396	37.3%	52	396	45.2%	51	388
CDC08	82.8%	51	396	27.6%	52	396	39.2%	51	388
CDC11	80.9%	51	396	2.45%	51	388	4.88%	50	375
CDC12	82.0%	51	396	6.93%	51	388	13.3%	51	388
CDC16	88.7%	51	396	1.64%	50	375	4.39%	51	388
CDC22	88.4%	51	396	2.71%	50	375	6.64%	51	388
CDC36	91.7%	52	396	7.44%	51	388	14.3%	51	388
CDC40	91.4%	51	396	13.9%	51	388	26.4%	51	388
CDC43	91.4%	51	396	29.3%	51	388	37.1%	51	388
CDC44	96.6%	50	380	7.44%	50	372	11.9%	50	372
CDC46	93.7%	51	396	0.727%	47	356	1.60%	50	375

^1^ Percentage of total sequence reads for the sample that matches the *Cyclospora cayetanensis* sequence. ^2^ The genotyping panel includes 396 SNP sites in 52 markers.

**Table 3:** Targeted amplicon sequencing and real-time PCR results for clinical fecal specimens and raspberries inoculated with clinical fecal specimens.

Sample	Inoculation	C_T_	%Ccay ^1^	Markers ^2^	SNPs ^2^
CDC04	NA	19.5 ± 0.02	80.7%	51	396
CDC04R1	50 uL undiluted	24.3 ± 0.1	73.0%	51	396
CDC04R2	50 uL of 1:10	25.3 ± 0.1	60.8%	51	396
CDC04R3	5 µL of 1:100	34.8 ± 0.3	64.8%	29	196
CDC16	NA	23.6 ± 0.05	88.7%	51	396
CDC16R1	50 uL undiluted	28.0 ± 0.4	72.2%	51	396
CDC16R2	50 uL of 1:10	28.4 ± 0.3	69.6%	51	396
CDC16R3	5 µL of 1:100	35.7 ± 1.5	73.6%	21	188
CDC43	NA	18.9 ± 0.1	91.4%	51	396
CDC43R1	50 uL undiluted	23.5 ± 0.1	68.9%	52	396
CDC43R2	50 uL of 1:10	23.6 ± 0.3	67.0%	52	396
CDC43R3	5 µL of 1:100	30.6 ± 0.2	73.8%	50	389

^1^ Percentage of total sequence reads for the sample that matches the *Cyclospora cayetanensis* sequence. ^2^ The genotyping panel includes 396 SNP sites in 52 markers.

## Data Availability

The data presented in this study are deposited in the NCBI Sequence Read Archive repository under BioProject PRJNA1052691 and assembled DNA sequences under BankIt2795740, BankIt2795744, and BankIt2795749.

## Supplementary Materials

The following supporting information can be downloaded via this link: https://www.mdpi.com/article/10.3390/microorganisms12050848/s1, Table S1: Number of different haplotypes observed in the specimens for each marker in the TAS panel; Figure S1: Hierarchical cluster dendrogram generated from a distance matrix including haplotyping results from the 66 clinical specimens included in this work and 40 WGS assemblies available at NCBI.

## References

[1] Mathison B.A., Pritt B.S. (2021). Cyclosporiasis-Updates on Clinical Presentation, Pathology, Clinical Diagnosis, and Treatment. Microorganisms.

[2] Chen Y., Qin Z., Li J., Xiao L., Zhang L. (2024). The global prevalence of *Cyclospora cayetanensis* infection: A systematic review, meta-analysis, and meta-regression. Acta Trop..

[3] Li J., Wang R., Chen Y., Xiao L., Zhang L. (2020). *Cyclospora cayetanensis* infection in humans: Biological characteristics, clinical features, epidemiology, detection method and treatment. Parasitology.

[4] Barratt J.L.N., Shen J., Houghton K., Richins T., Sapp S.G.H., Cama V., Arrowood M.J., Straily A., Qvarnstrom Y. (2023). *Cyclospora cayetanensis* comprises at least 3 species that cause human cyclosporiasis. Parasitology.

[5] Ortega Y.R., Sanchez R. (2010). Update on *Cyclospora cayetanensis*, a food-borne and waterborne parasite. Clin. Microbiol. Rev..

[6] Giangaspero A., Marangi M., Koehler A.V., Papini R., Normanno G., Lacasella V., Lonigro A., Gasser R.B. (2015). Molecular detection of *Cyclospora* in water, soil, vegetables and humans in southern Italy signals a need for improved monitoring by health authorities. Int. J. Food Microbiol..

[7] Chacin-Bonilla L., Santin M. (2023). *Cyclospora cayetanensis* Infection in Developed Countries: Potential Endemic Foci?. Microorganisms.

[8] Hadjilouka A., Tsaltas D. (2020). *Cyclospora Cayetanensis*-Major Outbreaks from Ready to Eat Fresh Fruits and Vegetables. Foods.

[9] Casillas S.M., Bennett C., Straily A. (2018). Multiple Cyclosporiasis Outbreaks—United States, 2018. Mmwr-Morbid Mortal W.

[10] Morton V., Meghnath K., Gheorghe M., Fitzgerald-Husek A., Hobbs J., Honish L., David S. (2019). Use of a case-control study and control bank to investigate an outbreak of locally acquired cyclosporiasis in Canada, 2016. Can. Commun. Dis. Rep..

[11] Kozak G.K., MacDonald D., Landry L., Farber J.M. (2013). Foodborne outbreaks in Canada linked to produce: 2001 through 2009. J. Food Prot..

[12] Yanta C.A., Pollo S.M.J., Barta J.R., Reiling S.J., Wasmuth J.D., Dixon B.R., Guy R.A. (2022). Draft Hybrid Genome Assembly of a Canadian *Cyclospora cayetanensis* Isolate. Microbiol. Resour. Announc..

[13] Qvarnstrom Y., Wei-Pridgeon Y., Van Roey E., Park S., Srinivasamoorthy G., Nascimento F.S., Moss D.M., Talundzic E., Arrowood M.J. (2018). Purification of *Cyclospora cayetanensis* oocysts obtained from human stool specimens for whole genome sequencing. Gut Pathog..

[14] Nascimento F.S., Barratt J., Houghton K., Plucinski M., Kelley J., Casillas S., Bennett C., Snider C., Tuladhar R., Zhang J. (2020). Evaluation of an ensemble-based distance statistic for clustering MLST datasets using epidemiologically defined clusters of cyclosporiasis. Epidemiol. Infect..

[15] Barratt J.L.N., Park S., Nascimento F.S., Hofstetter J., Plucinski M., Casillas S., Bradbury R.S., Arrowood M.J., Qvarnstrom Y., Talundzic E. (2019). Genotyping genetically heterogeneous *Cyclospora cayetanensis* infections to complement epidemiological case linkage. Parasitology.

[16] Houghton K.A., Lomsadze A., Park S., Nascimento F.S., Barratt J., Arrowood M.J., VanRoey E., Talundzic E., Borodovsky M., Qvarnstrom Y. (2020). Development of a workflow for identification of nuclear genotyping markers for *Cyclospora cayetanensis*. Parasite.

[17] Nascimento F.S., Barta J.R., Whale J., Hofstetter J.N., Casillas S., Barratt J., Talundzic E., Arrowood M.J., Qvarnstrom Y. (2019). Mitochondrial Junction Region as Genotyping Marker for *Cyclospora cayetanensis*. Emerg. Infect. Dis..

[18] Barratt J., Houghton K., Richins T., Straily A., Threlkel R., Bera B., Kenneally J., Clemons B., Madison-Antenucci S., Cebelinski E. (2021). Investigation of US *Cyclospora cayetanensis* outbreaks in 2019 and evaluation of an improved *Cyclospora* genotyping system against 2019 cyclosporiasis outbreak clusters. Epidemiol. Infect..

[19] Ahart L., Jacobson D., Rice M., Richins T., Peterson A., Zheng Y.L., Barratt J., Cama V., Qvarnstrom Y., Montgomery S. (2023). Retrospective evaluation of an integrated molecular-epidemiological approach to cyclosporiasis outbreak investigations—United States, 2021. Epidemiol. Infect..

[20] Rehme P. (2023). Notes from the Field: Doubling of Cyclosporiasis Cases Partially Attributable to a Salad Kit-Florida, 2021–2022. MMWR Morb. Mortal Wkly. Rep..

[21] Yanta C.A., Barta J.R., Corbeil A., Menan H., Thivierge K., Needle R., Morshed M., Dixon B.R., Wasmuth J.D., Guy R.A. (2022). Genotyping Canadian *Cyclospora cayetanensis* Isolates to Supplement Cyclosporiasis Outbreak Investigations. Microorganisms.

[22] Barratt J., Ahart L., Rice M., Houghton K., Richins T., Cama V., Arrowood M., Qvarnstrom Y., Straily A. (2022). Genotyping *Cyclospora cayetanensis* From Multiple Outbreak Clusters With An Emphasis on a Cluster Linked to Bagged Salad Mix-United States, 2020. J. Infect. Dis..

[23] Li J., Xu F., Karim M.R., Zhang L. (2022). Review on Cyclosporiasis Outbreaks and Potential Molecular Markers for Tracing Back Investigations. Foodborne Pathog. Dis..

[24] Leonard S.R., Mammel M.K., Gharizadeh B., Almeria S., Ma Z., Lipman D.J., Torrence M.E., Wang C., Musser S.M. (2023). Development of a targeted amplicon sequencing method for genotyping *Cyclospora cayetanensis* from fresh produce and clinical samples with enhanced genomic resolution and sensitivity. Front. Microbiol..

[25] Pightling A.W., Pettengill J.B., Luo Y., Baugher J.D., Rand H., Strain E. (2018). Interpreting Whole-Genome Sequence Analyses of Foodborne Bacteria for Regulatory Applications and Outbreak Investigations. Front. Microbiol..

[26] Barratt J.L.N., Plucinski M.M. (2023). Epidemiologic utility of a framework for partition number selection when dissecting hierarchically clustered genetic data evaluated on the intestinal parasite *Cyclospora cayetanensis*. Am. J. Epidemiol..

[27] Jacobson D.K., Low R., Plucinski M.M., Barratt J.L.N. (2023). An improved framework for detecting discrete epidemiologically meaningful partitions in hierarchically clustered genetic data. Bioinform. Adv..

[28] Qvarnstrom Y., Benedict T., Marcet P.L., Wiegand R.E., Herwaldt B.L., da Silva A.J. (2018). Molecular detection of *Cyclospora cayetanensis* in human stool specimens using UNEX-based DNA extraction and real-time PCR. Parasitology.

[29] Assurian A., Murphy H., Ewing L., Cinar H.N., da Silva A., Almeria S. (2020). Evaluation of the U.S. Food and Drug Administration validated molecular method for detection of *Cyclospora cayetanensis* oocysts on fresh and frozen berries. Food Microbiol..

[30] Balan K.V., Mammel M., Lipman D., Babu U., Harrison L.M., Almeria S., Durigan M., Leonard S.R., Hyein J., Gebru S. (2023). Development and Single Laboratory Evaluation of a Refined and Specific Real-time PCR Detection Method, Using Mitochondrial Primers (Mit1C), for Detection of *Cyclospora cayetanensis* in Produce. J. Food Prot..

[31] Maechler M., Rousseeuw P., Struyf A., Hubert M., Hornik K. (2022). Cluster: Cluster Analysis Basics and Extensions, R package Version 2.1.4.

[32] Bholowalia P., Kumar A. (2014). EBK-Means: A Clustering Technique based on Elbow Method and K-Means in WSN. Int. J. Comput. Appl..

[33] Rousseeuw P.J. (1987). Silhouettes—A Graphical Aid to the Interpretation and Validation of Cluster-Analysis. J. Comput. Appl. Math..

[34] Charrad M., Ghazzali N., Boiteau V., Niknafs A. (2014). Nbclust: An R Package for Determining the Relevant Number of Clusters in a Data Set. J. Stat. Softw..

[35] Balaban M., Moshiri N., Mai U., Jia X., Mirarab S. (2019). TreeCluster: Clustering biological sequences using phylogenetic trees. PLoS ONE.

[36] Markin A., Wagle S., Grover S., Vincent Baker A.L., Eulenstein O., Anderson T.K. (2023). PARNAS: Objectively Selecting the Most Representative Taxa on a Phylogeny. Syst. Biol..

[37] Korbie D.J., Mattick J.S. (2008). Touchdown PCR for increased specificity and sensitivity in PCR amplification. Nat. Protoc..

[38] Zhang Q., Wang J., Deng F., Yan Z., Xia Y., Wang Z., Ye J., Deng Y., Zhang Z., Qiao M. (2015). TqPCR: A Touchdown qPCR Assay with Significantly Improved Detection Sensitivity and Amplification Efficiency of SYBR Green qPCR. PLoS ONE.

[39] Gonzalez-Gomez J.P., Lozano-Aguirre L.F., Medrano-Felix J.A., Chaidez C., Gerba C.P., Betancourt W.Q., Castro-Del Campo N. (2023). Evaluation of nuclear and mitochondrial phylogenetics for the subtyping of *Cyclospora cayetanensis*. Parasitol. Res..

[40] Weitzel T., Brown A., Libman M., Perret C., Huits R., Chen L., Leung D., Leder K., Connor B.A., Menendez M.D. (2024). Intestinal protozoa in returning travellers: A GeoSentinel analysis from 2007 to 2019. J. Travel. Med..

[41] Herwaldt B.L., Ackers M.L. (1997). An outbreak in 1996 of cyclosporiasis associated with imported raspberries. The Cyclospora Working Group. N. Engl. J. Med..

[42] Temesgen T.T., Stigum V.M., Robertson L.J. (2022). Surveillance of berries sold on the Norwegian market for parasite contamination using molecular methods. Food Microbiol..

[43] Ho A.Y., Lopez A.S., Eberhart M.G., Levenson R., Finkel B.S., da Silva A.J., Roberts J.M., Orlandi P.A., Johnson C.C., Herwaldt B.L. (2002). Outbreak of cyclosporiasis associated with imported raspberries, Philadelphia, Pennsylvania, 2000. Emerg. Infect. Dis..

[44] Moreno-Mesonero L., Soler L., Amoros I., Moreno Y., Ferrus M.A., Alonso J.L. (2023). Protozoan parasites and free-living amoebae contamination in organic leafy green vegetables and strawberries from Spain. Food Waterborne Parasitol..

[45] Gonzalez-Ramirez L.C., Djabayan-Djibeyan P., Prato J.G., Garcia Rios C.A., Carrero J.C., Trelis M., Fuentes M.V. (2023). Field study of parasitic contamination of fruits, vegetables and leafy greens in the Ecuadorian Andes. F1000Research.

