# Supplementary material for: Evaluation of the Increased Genetic Resolution and Utility for Source Tracking of a Recently Developed Method for Genotyping Cyclospora cayetanensis
Source: Microorganisms. 2024 Apr 24;12(5):848. doi: 10.3390/microorganisms12050848 (PMC11124223; doi:10.3390/microorganisms12050848)
Supplement: Supplementary file 1 [file microorganisms-12-00848-s001.zip › microorganisms-2937128-supplementary.pdf]

Table S1. Number of different haplotypes observed in the set of 66 specimens for each of the 52 markers in the TAS panel.

Marker	Haplotypes
AA	2
AC	8
AD	6
AE	9
AF	8
AG	5
AH	3
AJ	3
AK	3
AL	4
AM	3
AO	8
AP	3
AQ	3
AR	3
AS	3
AU	3
AV	3
AW	3
AX	3
AY	3
AZ	5
CA	2
CB	2
CC	2
CD	2
CE	4
CF	5
CG	3
CH	4
CI	3
CJ	7
CK	3
FA	7
FB	9
FC	7
FD	10
FE	5
FF	4
FG	4

FH	8
FI	5
FL	4
FM	6
FN	8
FP	4
FQ	5
FR	3
FT	3
FU	4
FV	4
FW	3

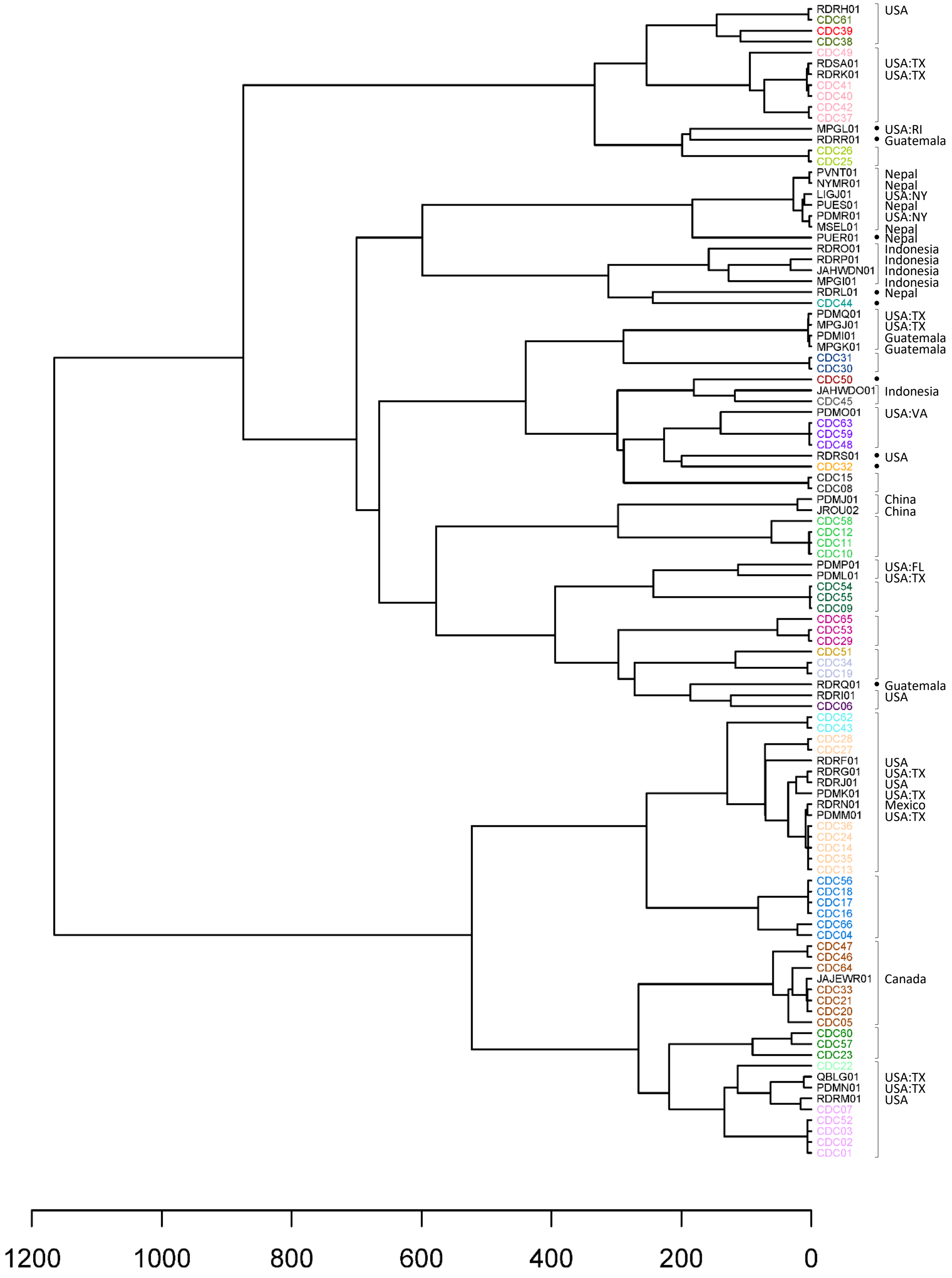

Figure S1. Hierarchical cluster dendrogram generated from a distance matrix including haplotype results from the 66 clinical specimens included in this study and 40 WGS assemblies available at NCBI. Accession numbers are used to label the WGS assemblies. The 31 clusters resulting from partitioning the dendrogram are denoted by either a bracket or by a dot for clusters containing a single sample. Font colors denote the specimens that cluster together based on partitioning the dendrogram without the WGS assemblies included (Figure 1A). Specimen collection locations are noted.
